# Mediastinal mature teratoma with complex rupture into the lung, bronchus and skin: a case report

**DOI:** 10.1186/1477-7819-11-125

**Published:** 2013-06-01

**Authors:** Mounia Serraj, Marouane Lakranbi, Jamal Ghalimi, Yassine Ouadnouni, Mohamed Smahi

**Affiliations:** 1 Faculty of medicine and pharmacy, Sidi Mohamed Benabdellah University, 30006 Fès Sidi Brahim, BP 5552, Fès, Morocco; 2 Department of Thoracic Surgery, University Hospital Hassan II, Km 2.200, Route de Sidi Harazem, BP 1893, 30000, Fez, Morocco; 3 Department of Respiratory Diseases, University Hospital Hassan II, Km 2.200, Route de Sidi Harazem, BP 1893, Fez, 30000, Morocco

**Keywords:** Mediastinal teratoma with airway communication, Skin fistula, Hemoptysis

## Abstract

Mature teratoma is the most common primary germ cell tumor in the mediastinum. On rare occasions, cystic teratomas rupture in adjacent structures, such as pleural space, pericardium, lung or tracheobronchial tree. We present a case of a mediastinal mature cystic teratoma in 16-year-old female with complex rupture into the lung, bronchus and skin. Mature mediastinal teratoma fistulized to the skin has not been previously described.

## Background

Mature teratoma is the most common primary germ cell tumor in the mediastinum [1]. It is composed of ectodermal, mesodermal, and endodermal derivatives. Typical computed tomography (CT) appearances are an encapsulated mass with a smooth wall containing soft tissue, fluid, fat, calcification or any combination of these [1].

These tumors rarely rupture into the adjacent structures, such as the pleural space, pericardium, lung parenchyma or tracheal tree. We report a case of mature mediastinal teratoma (MMT) with complex rupture into the lung, bronchus and skin with cysto-cutaneous fistula, which produced sebum and hair. To our knowledge, this observation has not previously been described.

## Case presentation

A 16-year-old female patient was admitted to the hospital with recurrent hemoptysis accompanied by chest pain for 5 months, and recent emergence of a left parasternal cutaneous fistula. She also complained of mild dyspnea on exertion and subfebrile body temperature.

**Figure 1:**
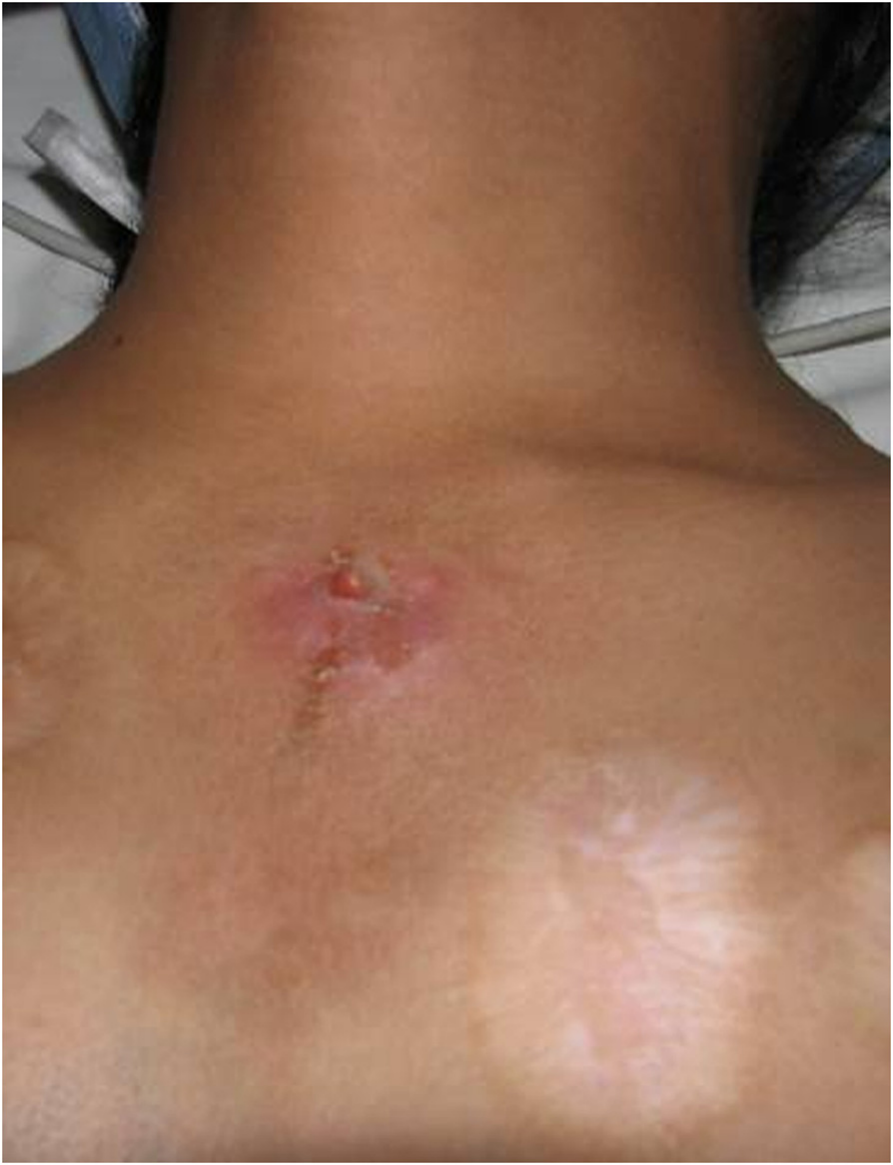
A left parasternal cutaneous fistula.

On admission, physical examination was normal except for the presence of a cutaneous fistula (Figure 1) at the level of the first left intercostal space between the parasternal and middle clavicular lines, surrounded by scars from skin burns due to ritual practices of traditional medicine. Yellowish sebaceous material flowed through the fistula, and this sometimes contained hair. Laboratory results showed no abnormalities.

**Figure 2:**
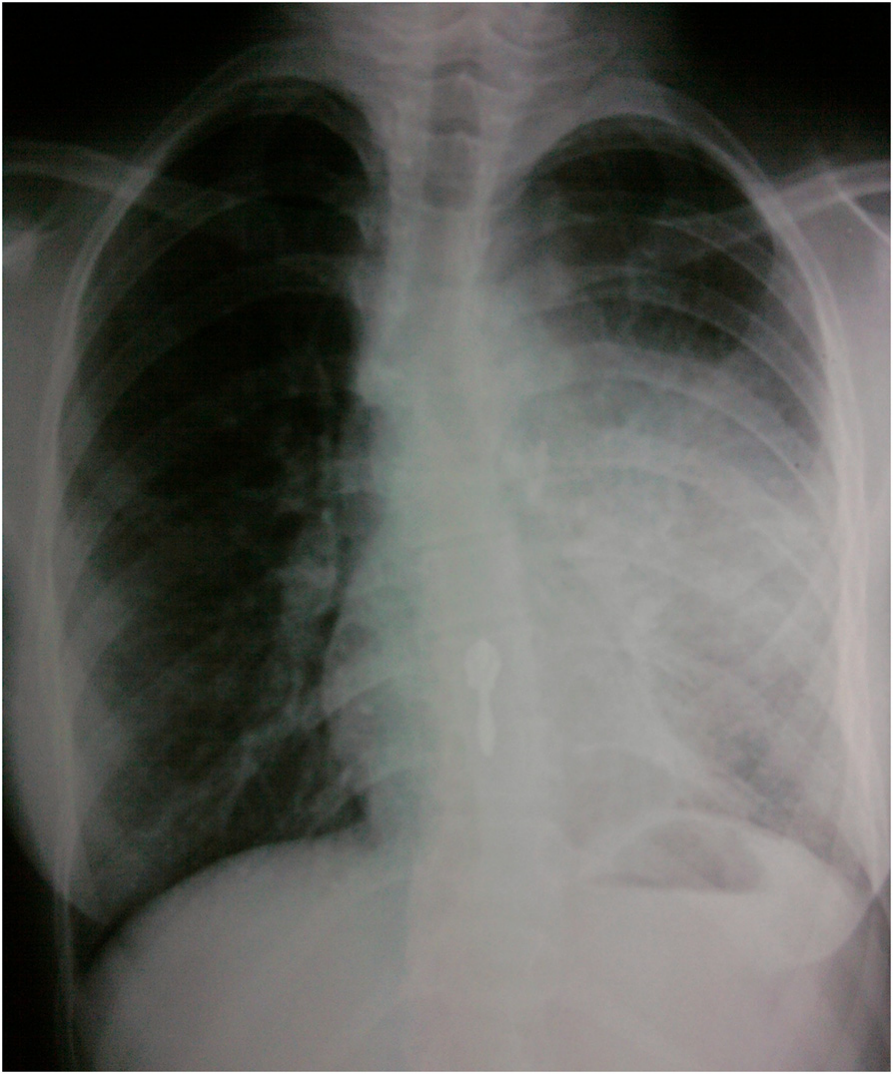
Chest radiography: left anterior mediastinal tumor.

**Figure 3:**
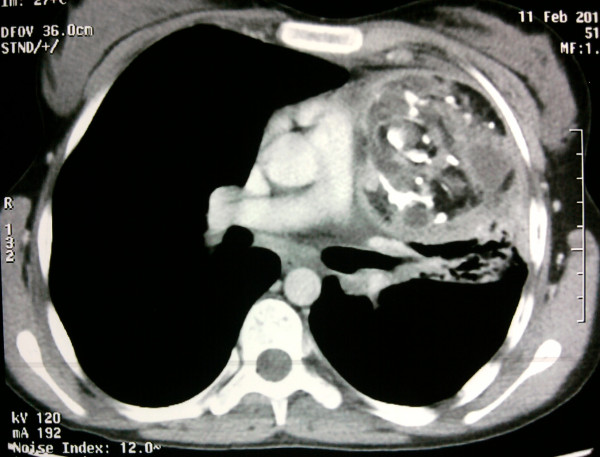
Contrast-enhanced computed tomography: heterogeneously enhanced mass with fatty and calcified components in the left anterior mediastinum infiltrating the left upper lobe, with pneumonitis and destruction of the lingula.

A simple thoracic radiograph showed a left anterior mediastinal mass (Figure 2). Contrast-enhanced CT revealed a heterogeneous mass with fatty and calcified components in the left anterior mediastinum, infiltrating the left upper lobe with pneumonitis and destruction of the lingula (Figure 3). Flexible bronchoscopy revealed hair-like structures at the orifice of the lingular bronchus.

MMT with complex rupture into the lung, bronchus and skin was suspected. Surgical treatment performed by a left posterolateral thoracotomy, consisted of complete tumor resection combined with resection of the lingula and flattening of the fistulous tract to the chest wall. The tumor was difficult to resect, and adhesions were noted. The tumor was removed with extreme care to preserve vital structures such as the phrenic nerve, recurrent laryngeal nerve, aorta, pulmonary and subclavian vessels.

**Figure 4:**
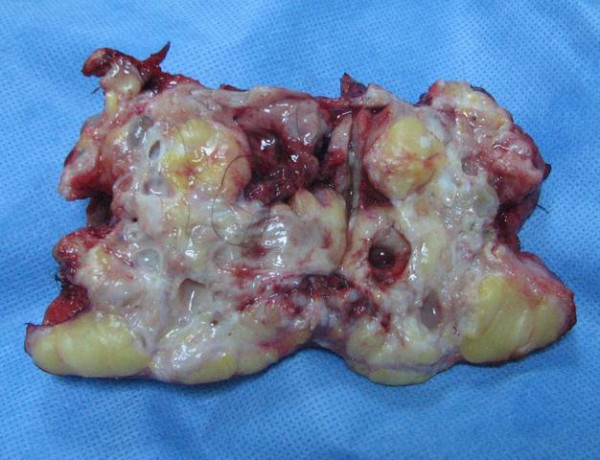
Surgical resection specimen.

The tumor contained yellowish sebaceous material as well as some hair and fat (Figure 4). Microscopy showed a cystic lesion lined by stratified squamous epithelium. There was adipose tissue, hair follicles, sweat glands, and sebaceous glands. A teratoma with a pulmonary fistula lined with fibrous tissue and an area of pneumonia were found. Perforation of the cystic teratoma was confirmed, and no immature component or malignancy was identified.

The postoperative period was uneventful and the patient was discharged on the sixth day after surgery. She remained well at 2-year follow up.

## Discussion

Mediastinal teratomas are asymptomatic in up to 53% of cases and are frequently discovered incidentally on chest radiography performed for other reasons [2]. Perforation of a mediastinal cystic teratoma is rare but is always symptomatic. Only a few publications report a high rate of teratoma rupture of 41.0% and 36.0% respectively [1,3].

The etiology of perforation of mediastinal teratomas is still controversial, although ischemia, infection and inflammation have been proposed [1]. Tumor size or wall thickness are not significant predisposing factors for tumor rupture [1]. On the other hand, cystic teratoma can produce proteolytic or digestive enzymes, leading to adhesion and erosion of surrounding structures [1]. The most common reason for rupture of benign mature mediastinalteratoma is autolysis caused by digestive enzymes released from pancreatic or salivary gland tissue of the tumor. As a result, a chronic inflammatory process in the cyst wall may cause rupture or fistula formation [1,3,4].

CT is the best modality to demonstrate tumor morphology and its complications. According to Choi *et al*. [1], in cases of mediastinal teratoma, CT findings of inhomogeneity of the internal components and changes in the adjacent lung parenchyma, pleura, or pericardium can be used as signs of tumor rupture. Cheung *et al*. [5] report the CT findings of a case of mediastinal cystic teratoma before and after rupture, and say that features of bursting of the spherical fatty component and intrapulmonary bronchial invasion, are also suggestive of rupture of the mediastinal teratoma. CT findings of ruptured cystic teratoma are important, not for early diagnosis, but also for surgical planning in determining the presence of tumor adhesion and infiltration of adjacent anatomical structures. Although atelectasis or pneumonitis account for most of the associated adjacent lung consolidation, intrapulmonary tumor infiltration is also possible.

Rupture of a mediastinal teratoma in an adjacent organ, including the lung, bronchus, pleura, and pericardium is classic, and is often reported as a clinical case. To our knowledge, complex rupture of a mediastinal teratoma simultaneously in the lung, bronchi and skin has never been described, this is the first case reported in the literature. In this case, the existence of a cutaneous fistula with flow of sebum containing hair, is like trichoptysis, pathognomonic of ruptured mediastinal teratoma.

Prompt treatment of mediastinal teratoma is necessary to confirm the diagnosis by eliminating the presence of an immature component, and avoid complication due to its rupture causing hemoptysis, acute respiratory distress or cardiac tamponade [6]. Surgery on a mediastinal teratoma is often complicated by tight adhesions to vital mediastinal structures such as the phrenic nerve, aorta, vena cava, and pulmonary and supra aortic vessels [6], and is even more so with a ruptured teratoma because of the inflammation and infiltration [7].

## Conclusions

In mature mediastinal cystic teratoma, even if the fistula to the skin and lung is an unusual and anecdotal complication, reflecting its long history and its late diagnosis, this is further evidence of the benefits of performing surgery without delay, and confirming the diagnosis by eliminating the presence of immature or malignant components, and thus avoiding complications.

## Consent

Written informed consent was obtained from the patient’s parent for the study and publication of this case report and accompanying images. A copy of written consent is available for review from the Editor-in-Chief.

## Abbreviations

CT: Computed tomography; MMT: Mature mediastinal teratoma.

## Competing interests

The authors declare that they have no competing interests.

## Authors’ contributions

SM collected information and prepared the original draft. LM and GJ researched the relevant literature and revised the draft. OY helped with the literature research and preparing the manuscript. SM helped prepare the manuscript. All authors read and approved the final manuscript.
